# Toward Diabetes Device Development That Is Mindful to the Needs of Young People Living With Type 1 Diabetes: A Data- and Theory-Driven Qualitative Study

**DOI:** 10.2196/43377

**Published:** 2023-01-25

**Authors:** Nicola Brew-Sam, Anne Parkinson, Madhur Chhabra, Adam Henschke, Ellen Brown, Lachlan Pedley, Elizabeth Pedley, Kristal Hannan, Karen Brown, Kristine Wright, Christine Phillips, Antonio Tricoli, Christopher J Nolan, Hanna Suominen, Jane Desborough

**Affiliations:** 1 National Centre for Epidemiology and Population Health, College of Health and Medicine The Australian National University Canberra Australia; 2 Philosophy Section, Faculty of Behavioural, Management, and Social Sciences University of Twente Enschede Netherlands; 3 The Centenary Hospital for Women and Children Canberra Health Services Canberra Australia; 4 School of Medicine and Psychology, College of Health and Medicine The Australian National University Canberra Australia; 5 Nanotechnology Research Laboratory, Faculty of Engineering The University of Sydney Sydney Australia; 6 Nanotechnology Research Laboratory, Research School of Chemistry, College of Science The Australian National University Canberra Australia; 7 The John Curtin School of Medical Research, College of Health and Medicine The Australian National University Canberra Australia; 8 Department of Endocrinology and Diabetes Canberra Health Services Canberra Australia; 9 School of Computing, College of Engineering, Computing and Cybernetics The Australian National University Canberra Australia; 10 Department of Computing University of Turku Turku Finland

**Keywords:** type 1 diabetes mellitus, unified theory of acceptance and use of technology, UTAUT, value-sensitive design, young people, data- and theory-driven analysis, improved device design

## Abstract

**Background:**

An important strategy to understand young people’s needs regarding technologies for type 1 diabetes mellitus (T1DM) management is to examine their day-to-day experiences with these technologies.

**Objective:**

This study aimed to examine young people’s and their caregivers’ experiences with diabetes technologies in an exploratory way and relate the findings to the existing technology acceptance and technology design theories. On the basis of this procedure, we aimed to develop device characteristics that meet young people’s needs.

**Methods:**

Overall, 16 in-person and web-based face-to-face interviews were conducted with 7 female and 9 male young people with T1DM (aged between 12 and 17 years) and their parents between December 2019 and July 2020. The participants were recruited through a pediatric diabetes clinic based at Canberra Hospital. Data-driven thematic analysis was performed before theory-driven analysis to incorporate empirical data results into the unified theory of acceptance and use of technology (UTAUT) and value-sensitive design (VSD). We used the COREQ (Consolidated Criteria for Reporting Qualitative Research) checklist for reporting our research procedure and findings. In this paper, we summarize the key device characteristics that meet young people’s needs.

**Results:**

Summarized interview themes from the data-driven analysis included aspects of self-management, device use, technological characteristics, and feelings associated with device types. In the subsequent theory-driven analysis, the interview themes aligned with all UTAUT and VSD factors except for one (privacy). Privacy concerns or related aspects were not reported throughout the interviews, and none of the participants made any mention of data privacy. Discussions around ideal device characteristics focused on reliability, flexibility, and automated closed loop systems that enable young people with T1DM to lead an independent life and alleviate parental anxiety. However, in line with a previous systematic review by Brew-Sam et al, the analysis showed that reality deviated from these expectations, with inaccuracy problems reported in continuous glucose monitoring devices and technical failures occurring in both continuous glucose monitoring devices and insulin pumps.

**Conclusions:**

Our research highlights the benefits of the transdisciplinary use of exploratory and theory-informed methods for designing improved technologies. Technologies for diabetes self-management require continual advancement to meet the needs and expectations of young people with T1DM and their caregivers. The UTAUT and VSD approaches were found useful as a combined foundation for structuring the findings of our study.

## Introduction

### Background

Type 1 diabetes mellitus (T1DM) is an autoimmune condition often diagnosed in children and adolescents [1]. It requires lifelong self-management, including blood glucose monitoring; adherence to insulin regimens; lifestyle adjustments, including diet and exercise; and, for many, the management of psychological health [2]. Advanced diabetes technologies, such as insulin pumps, continuous glucose monitors (CGMs), and closed loop systems, have been found to improve self-management and quality of life among young people with diabetes [3]. Moreover, the use of such technologies can help reduce the risk of acute and long-term diabetes complications [4].

An important strategy to understand young people’s needs and preferences regarding technologies for T1DM management is the examination of their day-to-day experiences with these technologies. The analysis of their experiences with technologies also serves to identify their specific perceptions, decisions, and behaviors regarding technology use [5,6]. Co-design research is enriched through feedback on experiences with technology use [7]. The experiences of young people with T1DM with diabetes technologies have been studied mainly using exploratory research designs [8,9].

Exploratory (qualitative) research (eg, into user experiences) is distinguished by the absence of a priori theory in social sciences; it is an inductive process that is used to generate knowledge or theory [10]. By contrast, technology adoption and use are predicted and explained using sociotechnical theories in social sciences and computer science (such as technology acceptance models), which informs technology design decisions. Research on both user experience and technology acceptance ultimately aims to understand the mechanisms that shape the uptake and use of technology [11]. It is only recently that the merit of combining both approaches has been recognized, for example, by investigating how user experiences can inform technology acceptance models [12] or how knowledge from technology acceptance models can advance or structure user experience research [13]. The latter can be implemented using a transdisciplinary theory-driven analysis of exploratory qualitative empirical data [13]. A stronger focus on theory can strengthen and advance the translational outcomes of research [14,15], whereas a greater focus on exploratory inductive research can deepen the validity and applicability of findings. In the case of user experience research, technology acceptance and technology design theories provide the opportunity to align a data-driven approach with a theory-driven analysis. Incorporating the theoretical framework of technology acceptance and technology design knowledge into the study of the experiences and preferences of young people regarding diabetes technologies can promote a well-grounded foundation for future research in this area and can inform the development of improved diabetes technologies for young people on a sound basis.

### Study Aim

Our study had 3 aims. The first aim was to examine young people’s and their caregivers’ experiences and preferences regarding insulin pumps, sensor technologies, and diabetes communication technologies in an exploratory manner. The second aim was to relate the findings to selected technology acceptance and technology design theories. The third aim was to develop, based on the outcomes of the second aim, device characteristics that would meet the needs of young people. Thus, we aimed to highlight the benefits of incorporating validated theory into empirical user experience research for designing new and improved technologies.

## Methods

### Overview

We conducted 16 interviews with young people with T1DM and their parents about their use of diabetes technologies. The interviews were conducted face to face, either in person or on the web, by female academic health experience researchers (AP, JD, MC, and NB-S; PhD or MPH degree; Health Experience Team of *Our Health in Our Hands* at the Australian National University) with experience in conducting qualitative research. Interviews were held between December 2019 and July 2020 (12 in-person interviews and 4 web-based video calls owing to the onset of COVID-19) until data saturation was reached. The participants were recruited through a pediatric diabetes clinic based at Canberra Hospital. The first contact was established either at appointments at the clinic through invitation from pediatric endocrinologists, or in response to a study flyer in the waiting room, or through invitations sent via email by the study coordinator. There was no prior relationship between the participants and the interviewers. The interviews lasted between 20 and 30 minutes. In-person face-to-face interviews were conducted at the National Center for Epidemiology and Population Health at the Australian National University. After 16 interviews, preliminary data analysis showed that we had reached thematic saturation. The sample size was similar in magnitude to the qualitative studies included in a previous review on the experiences of young people and their caregivers with diabetes technology use [8], although the length of the interviews in our study was shorter (an average of 20 to 30 minutes) than that in the studies in the review (20 minutes to 2 hours). The interview protocol was carefully designed to elicit the desired information. Although some participants were less talkative than others, overall, we were able to gain sufficient data to inform the study. No major differences were noted between the in-person and web-based interviews.

The interview protocol focused on exploratory data collection for a data-driven analysis, which was the first step, without the influence of preexisting categories, whereas, as the second step, a theory-driven analysis was performed to enable the alignment of the interview themes with the categories from the selected theories. To achieve this, the interview protocol was kept as open-ended and short as possible, with general probing questions to initiate conversation about experiences with diabetes technologies and related preferences. It contained questions about managing diabetes with technological devices, the types of devices used and preferred, experiences with these devices, decision-making, and the challenges encountered. The interview protocol was developed in collaboration with 3 young people with T1DM who were members of our research team (EB, KH, and LP). The interview probes (questions) were first drafted by the researchers, then discussed with the young people (EB, KH, and LP) and other research partners (pretest), and, finally, revised to make them more concise and clearer for the participants. All the interviews were audio recorded and professionally transcribed.

### Ethics Approval

Ethics approval was obtained from the Australian National University’s Human Ethics Committee and the Australian Capital Territory Health Human Research Ethics Committee in October 2019 (2019.ETH.00143 and 2019/ETH121700). In addition, an ethics protocol variation for web-based interviews was approved by the same committees in April 2020. All the interviews were conducted with informed consent.

### Data Analysis

For data analysis, we used a combination of data-driven thematic analysis (stage 1) and theory-driven analysis (stage 2) to pay respect to rich data generated from both the interviews and the existing literature. Conducting our data-driven analysis before the theory-driven analysis enabled the identification of themes without the influence of theoretical factors, ensuring that the analysis was not limited to theoretical factors.

Stage 1 followed a qualitative data-driven thematic analysis approach based on Braun and Clarke [16]—data familiarization and coding, generation of themes, thematic review, definition of themes, and reporting—to identify themes arising from the 16 interviews. Transcripts were uploaded to the NVivo (version 12, QSR International) data management software. Two researchers (MC and NB-S) familiarized themselves with the data via multiple readings; then, codes were identified from the keywords and phrases of interest and compared and combined to form the coding schema for the data-driven analysis (Multimedia Appendix 1). Ongoing discussions between the 4 researchers (AP, JD, MC, and NB-S) were held throughout the data analysis process to ensure construct validity. We did not perform an internal comparison of different emerging themes in relation to their frequency of endorsement. Our goal was not such a quantitative weighing of themes but an inclusive search for thematic breadth from the interviews.

In the second stage, the same researchers analyzed the initial themes from the interviews using selected relevant theories on (1) technology acceptance and use and (2) technology design by classifying and sorting the interview themes and results into theoretical factors. We collected all the interview themes and excerpts that represented, for example, *accessibility issues* and reported them alongside the technology design factor *accessibility* (refer to the selected theories and factors in Tables 1 and 2).

**Table 1:** Explanation of the unified theory of acceptance and use of technology (UTAUT) factors [17] and their alignment with interview data and systematic review [8].

Model, category, and factor				Factor explanation or definition [17]		Interview data theme		Relevant interview data excerpt		Relevant systematic review theme [8]
**UTAUT**										
	**Core determinant**									
		Effort expectancy	The ease of the technology use		Ease of and effort needed for use (CGM^a^, FGM^b^, and pump)		IP^c^4: “you just wipe it [FGM] and you get the number”		Discussed as a part of impact on blood glucose control	
		Facilitating conditions	Infrastructure (eg, organizational or technical) supporting the technology use		HCP^d^ support, support at school and at home, customer service of technology, and programs and funding (CGM and pump)		IP4: “we can call them [health care team] at any time of the day”		Impact on independence and relationships	
		Performance expectancy	The extent to which the user and nonuser believes that using the technology will improve their performance		Expectations regarding self-management with technology: device expectation, success in self-management, and preferences (CGM and pump)		IP1: “satisfaction of seeing it [blood glucose management] successful”		Impact on blood glucose control and satisfaction with technologies	
		Social influence	The degree to which someone thinks that it is important others believe that they should (not) use the technology		Influence on technology use (parents, peers, and HCPs), technology suggested by physician, and child’s or parents’ decision to use technology (age dependent)		IP1P: “[physician name] sort of seems to be quite keen on devices and quite keen on pumps”		Impact on independence and relationships	
	**Moderator**									
		Gender and age	The user and nonuser’s gender and age		Gender and age differences		IP1: “all the kids have Dexcom [CGM], my age”		Experiences with alarms, satisfaction with the technologies, and age (groups) as a general background variable	
		Use experience	Previous experience with the technology		Length of disease and length of technology use		IP3P: “now that it’s part of our lives [diabetes], I’m very grateful for my experiences”		Across all themes	
		Voluntariness of use	The degree to which it is perceived that the technology is used out of free will		Influence on technology use (parents, peers, and HCPs), technology suggested by physician, and child’s or parents’ decision to use technology (age dependent)		IP16P: “I don’t want to force [child] [to use technology] since it’s [child’s] body”		Impact on independence and relationships	

^a^CGM: continuous glucose monitor.

^b^FGM: flash glucose monitor.

^c^IP: interview participant identifiers for young people (eg, IP1) and their parents (eg, IP1P).

^d^HCP: health care professional.

**Table 2:** Explanation of value-sensitive design (VSD) factors [18] and their alignment with interview data and systematic review [8].

Model, category, and factor				Factor explanation or definition [18]		Interview data theme		Relevant interview data excerpt		Relevant systematic review theme [8]
**VSD**										
	**System feature**									
		Connectivity	Features that allow the user to interact with the devices and share information with others		Connectivity among CGM^a^, pump, watch, and apps; data sharing and access (HCPs^b^ and family); and downloading before sharing		IP^c^14P: the “devices [CGM, pump, apps, etc]...talk to each other”		Impact on independence and relationships: data sharing Device design and features: connectivity and calibration	
		Data analysis	Features that allow the user to make sense of data over time		Data trends and graphical outputs (display; CGM and pump)		IP14P: “not always a hundred percent accurate” (using CGM data to calculate HbA1c^d^)		Device design and features: data trends	
		Data retrieval and storage	Features that allow the user to access and store data		Apps and web-based sources for information, cloud storage, and storage in devices (blood glucose meter and pump)		IP6: “and the pump...it stores all the information that you have”		Data access discussed in terms of its impact on sleep and overnight experiences (eg, sensors)	
	**Value**									
		Accessibility	The system’s availability, adaptability, and portability		Technology adaption to new situations and conditions (eg, travel, sports, camp, sleepover, and night; regarding alarms, tape, size etc) and data accessibility in these situations (CGM and pump)		IP1P: “having it [CGM] meant [child] could go to school camp”		Impact on sleep and overnight experiences Experiences with alarms Device design and features: discomfort	
		Accountability and autonomy	Self-responsibility for habits and care performance, with independent behavior and decision-making		Increased self-responsibility, independence from parents, sense of control, and interference of parents (CGM, FGM^e^, and pump)		IP3P: “[child] doesn’t really want to have [child’s] parents knowing what [child is] doing all the time”		Impact on independence and relationships	
		Compliance	Adherence, following the diabetes care plan		Self-management compliance (style of management, including blood glucose testing, medication, etc)		IP1 or IP1P: “tend to over worry, and overly focus [on diabetes care]”		Impact on blood glucose levels (better management decisions with technology) Impact on sleep and overnight experiences (improved management at night) Experiences with alarms (affect compliance at school)	
		Dignity	Sense of pride and self-respect (impacted by negative outcomes or unfair treatment for performance)		Dignity: discrimination and unfair treatment (school)		IP3P: “you sort of feel like there’s this constant discrimination for something that [child] has no control over” (diabetes)		Impact on independence and relationships Experiences with alarms (school)	
		Empathy	Desire to be understood by others		Empathy: shown by friends, family, and HCPs		IP5P: “they [siblings] were all lining up for finger pricks” (empathy)		Impact on independence and relationships	
		Feedback	Responses from others or technology		Feedback: from HCPs and parents (CGM and pump)		IP15P: “it [CGM] just constantly alarmed for everything”		Impact on independence and relationships Experiences with alarms	
		Hope and joy	Motivation to meet future-oriented expectations and personally valued goals, including joy in life		Hope to meet the self-management goals with technology and increasing joy with technology, for example, through anxiety alleviation (CGM and pump)		IP15P: “sometimes [child] likes to have a break” (hope for normality and enabling joy)		Expectations before technology use	
		Privacy	Information protection when sharing sensitive (health) data		Not mentioned in interviews		Not mentioned in interviews		Not part of the themes	
		Sense making	Ability to give meaning to data		Sense making of data and understanding data based on diabetes education; graphical outputs were helpful		IP16P: “we were learning so much about diabetes”		Device design and features: trends and graphs	
		Trust	Trust in technology, oneself, and others		Trust in body (confidence in self and hypo awareness) versus technology (accuracy, technology failures, time lag, and reliability; CGM and pump), and trust in HCP team		IP14: “sometimes it [CGM] gets very inaccurate”		Device design and features: data lag	

^a^CGM: continuous glucose monitor.

^b^HCP: health care professional.

^c^IP: interview participant identifiers for young people (eg, IP1) and for parents (eg, IP1P).

^d^HbA1c: hemoglobin A1c.

^e^FGM: flash glucose monitor.

Both technology acceptance and technology design theories are highly relevant as a foundation for analyzing experiences with diabetes technologies to develop device characteristics that meet the needs of users. Technology acceptance and use approaches posit factors that attempt to explain or predict use intentions and decisions, whereas technology design approaches posit factors for improved technology design. In our study, these theoretical branches tackled different sides of the same coin (improving technology and its uptake) and were able to complement each other to provide a broader picture. Thus, an integration of knowledge from both approaches offered comprehensive guidance for our analysis of diabetes technology user experiences. In the following sections, we further explain which specific theories we selected and how they complemented each other.

### Theoretical Foundations for Theory-Driven Analysis

Technology acceptance models offer a sound framework for examining decisions and behaviors regarding health technology use. The unified theory of acceptance and use of technology (UTAUT) comprises elements from 8 previously well-established models [17] and has been applied in recent studies examining the acceptance of information and communication technologies by patients with diabetes [19,20] and health care professionals [21]. The UTAUT summarizes 8 factors that directly or indirectly influence technology use intention or use behaviors [17]. It comprises 4 core determinants of technology use intention and actual use—performance expectancy, effort expectancy, social influence, and facilitating conditions—and 4 additional factors—gender, age, use experience, and voluntariness of use—that act as moderators. For example, previous experiences with technologies moderate associations between other antecedent factors and use intention [17] or technology use [22] and influence technology uptake [23,24]. For a full description of these factors, refer to Table 1.

However, technology acceptance models such as UTAUT are subject to certain limitations owing to their binary logic of technology acceptance (acceptance or rejection) [25] and their assumption of the underlying rational behavior [26], which are at odds with the principles of a complex self-managing ecosystem in which users with varying needs, desires, and interests make decisions and act within a sociocultural context [18]. This is where our selected technology design approach can fill a gap and complement UTAUT for developing device characteristics that meet young people’s needs.

Value-sensitive design (VSD) offers a holistic methodological framework that integrates users’ values and life circumstances into the examination of their interaction with technologies [27]. It is underpinned by an integrative and iterative methodology that consists of 3 interrelated yet distinct investigations: technical investigations that focus on the technology, empirical investigations that gather the responses of individuals or groups affected by the technology, and conceptual investigations that examine the values of key stakeholders [27]. VSD is used to identify and conceptualize users’ values and to design technologies in accordance with these values [18]. For example, technically viable implantable medical devices can be undesirable for some patients and not align with their values [28]. In their study of adults with type 1 or type 2 diabetes, Dadgar and Joshi [18] used a VSD lens to identify system features and values that are important to people with diabetes to complement the usual functionalist approach to usability. They summarized 4 system features—connectivity, data analysis, data retrieval, and storage—and 12 values—accessibility, accountability and autonomy, compliance, dignity, empathy, feedback, hope and joy, privacy, sense making, and trust (extended to include technology)*—*relevant to the design of diabetes technologies. For a complete description of these features and values, refer to Table 2.

For example, Dadgar and Joshi [18] found that *trust* in technologies affects their use by patients, with a lack of trust in the devices leading to technology resistance and masking the advantage of the respective technology. The value *trust* also includes trust in others who use technologies to provide care. The authors acknowledge that patients’ technology use is embedded in self-management activities and relationships with family, friends, and health care providers. As self-management is integral to the well-being of people with diabetes and technology is rapidly developing in this space, marrying the needs of users with technology design is essential. Although VSD has been applied to the design of technologies for children and youth [29], a specific summary of values and system features focusing on young people with diabetes could not be found, and the work of Dadgar and Joshi [18] provided a useful foundation on which to build.

Using both theoretical approaches in the second part of our data analysis, we assessed the alignment of our initial themes with key factors from UTAUT and VSD. There was little overlap between the factors in the UTAUT and the selected VSD approach because of the different focuses of the theories, and thus, all factors from both theories could be used to guide the analysis. In Tables 1 and 2, we provide definitions of the respective factors and the alignment of the data with the theoretical factors. Multimedia Appendix 2 [18,27,30-33] provides a working example of how the VSD factors were adapted from the work of Friedman et al [27] and Dadgar and Joshi [18], and Multimedia Appendix 3 [17,18] presents data excerpt examples.

### Summary of Device Characteristics Meeting Young People’s Needs

On the basis of the analysis, the device characteristics that meet the needs of young people with T1DM were summarized. Key points and major themes from the data-driven analysis were collected alongside the theoretical factors to describe expectations, preferences, and needs, as articulated in the interviews. We did not focus on a quantitative summary but rather on important and highlighted aspects that emerged throughout the interviews regarding the device characteristics. For example, the interview participants (IPs) highlighted the importance of device accuracy and reported problems with time lags and technical failures, which affected their trust in the devices. Thus, the accuracy and reliability of the devices were summarized as important device characteristics aligned with the VSD factor *trust*.

Throughout the reporting of our study methods and results, we used the COREQ (Consolidated Criteria for Reporting Qualitative Research) checklist, as presented in Multimedia Appendix 4. The study results were presented to and discussed with the Health Experience Team of *Our Health in Our Hands* at the Australian National University, which included young people with T1DM and medical researchers.

## Results

### Study Sample and Devices Used

The sample included 16 young people with T1DM (female: n=7, 44%; male: n=9, 56%) aged between 12 and 17 years and accompanied by a parent (mother: n=11, 69%; father: n=4, 25%; both: n=1, 6%). We focused on this age group because of 3 reasons. First, government subsidies are limited to those aged <21 years [34]. Second, younger individuals have been reported to be “more exposed to new technologies and easier to absorb the new technological advancements with minimum effort” [35]. Third, adolescence—with a transition from childhood to adulthood—is accompanied by general life challenges affecting diabetes management [36,37]. The young people in our study had been diagnosed with T1DM between 1 and 14 years before this study.

Overall, 81% (13/16) of participants used an insulin pump (t:slim [Tandem Diabetes Care Inc]: n=3, 23%; Medtronic: n=10, 77%), 88% (14/16) used a CGM (Dexcom: n=11, 79%; Guardian [Medtronic plc]: n=3, 21%), and 6% (1/16) used a flash glucose monitor (FGM; FreeStyle Libre [Abbott Laboratories]). The participants used an insulin pump (2/16, 12%), a CGM (3/16, 19%), or a combination of both (11/16, 69%). In one case the FGM system was used in addition to the pump. Additional devices used included the Apple watch (Apple Inc; previously used: 2/16, 12%; currently using: 1/16, 6%; planned to use: 1/16, 6%), diabetes apps on smartphone or smart devices (various), and glucose and ketone meters (16/16, 100%). An overview of the study sample is presented in Table 3.

**Table 3:** Study sample overview^a^.

IP^b^	Mode	Sex	Year of diagnosis (range)	CGM^c^	Insulin pump	Other technology
1	Face to face	Male	2016-2020	Dexcom	No pump	—^d^
2	Face to face	Female	2011-2015	Dexcom	t:slim (Tandem Diabetes Care Inc*)*	Previously, Apple watch (Apple Inc)
3	Face to face	Male	2011-2015	No CGM and previously, Dexcom	Medtronic	—
4	Face to face	Male	2016-2020	Dexcom	t:slim	FreeStyle Libre (Abbott Laboratories**)** and thinking about Apple watch
5	Face to face	Male	2006-2010	Dexcom	Medtronic	—
6	Face to face	Male	2006-2010	Dexcom	Medtronic	Apple watch
7	Face to face	Female	2006-2010	Dexcom	Medtronic	—
8	Face to face	Female	2016-2020	No CGM	Medtronic	—
9	Face to face	Male	2006-2010	Medtronic (Guardian, Medtronic plc)	Medtronic	—
10	Face to face	Female	2006-2010	Dexcom	Medtronic	—
11	Face to face	Male	2011-2015	Dexcom	Medtronic	—
12	Face to face	Female	2016-2020	Dexcom	t:slim	—
13	Zoom (Zoom Video Communications)	Male	2011-2015	Medtronic (Guardian)	Medtronic	Previously, Dexcom and Apple watch
14	Zoom	Female	2016-2020	Dexcom	No pump yet; t:slim planned	—
15	Zoom	Male	2006-2010	Medtronic (Guardian)	Medtronic	—
16	Zoom	Female	2016-2020	Dexcom	No pump	—

^a^The participants were aged between 12 and 17 years; each young participant was accompanied by a parent or parents.

^b^IP: interview participant identifier for young people (eg, IP1).

^c^CGM: continuous glucose monitor.

^d^Not available.

### Interview Themes and Alignment With Theoretical Factors

Initial themes (Multimedia Appendix 1) identified from the interview data included information related to (1) sociodemographic characteristics, (2) medical diabetes and diabetes self-management (eg, diagnosis, family members with diabetes, diabetes education, hypoglycemic or hyperglycemic awareness and events, and style of self-management), (3) device use (eg, types of devices used, length and frequency of use, and preferences), and (4) specific device type–related technological characteristics and feelings associated with the specific device type used (CGM, insulin pump, or FGM). Themes were mostly related to the use of CGM, insulin pump, or a combination of both. Cases where the themes were related to the use of FGM or other devices have been specifically mentioned in the result section on UTAUT factors or VSD factors. Tables 1 and 2 also specifically mention the device types that the themes are related to.

In the following sections, the findings focus on the alignment of the initial themes with the UTAUT and VSD key factors (Tables 1 and 2) and are reported in accordance with these factors (data excerpts in Multimedia Appendix 3). The participants’ statements are cited with interview participant identifiers IP (eg, young interview participant 1 has been referred to as IP1), and their parents’ statements are indicated with an additional *P* following the number (eg, the parent of the young IP1 has been referred to as IP1P).

### UTAUT Factors

#### Performance Expectancy

The participants described how CGM and pump technologies contributed to their success in T1DM self-management and how the use of the devices made self-management easier. CGM improved and facilitated blood glucose tracking (IP14 and IP16), allowed the young people to take breaks from diabetes management (IP13P), and led to the *“*satisfaction of seeing it [blood glucose management] successful*”* (IP1). The direct connection of CGMs to phones was considered a major benefit (IP7). The insulin pump assisted in stabilizing blood glucose levels (IP14 and IP15), reduced the use of needles (IP2 and IP6), allowed flexible eating (IP2, IP6, and IP15), and was viewed as convenient and a device that enables a normal life (IP5P). The reduction in the use of injections by using a pump was perceived as a major advantage for small children (IP5P). Avoiding calculations was considered another benefit of the insulin pump (IP12P). Most young people expressed appreciation for having the devices, and this was also highlighted by one of the parents (IP6P).

#### Effort Expectancy

CGM was perceived as easy to use and put on (IP16). It did not require constant *“*fiddling*”* (IP13) and saved time (IP1 and IP5). The phone could be quickly checked instead of using finger pricks (IP2, IP6, IP7, IP11, and IP12). In addition, FGM was perceived as easy to use because *“*you just swipe it and you get the number*”* (IP4), as was the insulin pump (IP8P). The participants mentioned that they had to *“*calculate the carbs*”* for calculating the insulin dose delivered through the pump (IP3, IP4P, and IP8P). *“*When you just click that, so you can bolus there, put a basal on, like all that cool stuff*”* (IP7). The pump was considered easier to handle than insulin pens (IP5P, IP6, and IP9), owing to data storage options (IP6) and “pre-programmed” calculations (IP3):

I think the biggest thing when [name] got the pump for us as a family, it has made it much easier because...on a long car journey, we’ve got two older children, so they’d be constantly “can we have some food?” And I’d be like “no, because [name] has got to have another injection.” So once he got the pump, “yeah, sure you can have some. Just dial up some more insulin, [name].”

IP6P

Difficulties in charging devices in some situations were mentioned (IP6P), which led to the use of insulin pens in certain circumstances (IP6P). However, in most situations, the pump could be used (refer to *Accessibility*).

#### Social Influence and Voluntariness of Use

Physicians recommended CGMs (IP6 and IP14) and insulin pumps (IP1P, IP2P, and IP16P), whereas people with T1DM paid particular attention to the devices used by their peers with T1DM (IP14). This helped them make decisions regarding their own devices (IP16):

I ask them about their pumps, because they’ve all got the same pump...so I talk to them about it and try and get, like, what they think about it.

IP14

Social influence on device decisions was closely related to the voluntariness of use, reflecting the degree to which technology use was perceived to be volitional [17]. Device decisions were taken by parents for very young children (IP10P), whereas adolescents reported that they made informed decisions on their own or together with their parents (IP3P). The parents of all the participants were closely involved in diabetes management, as the participants were all minors. Nevertheless, some young people decided against insulin pumps (IP1 and IP16) even though their physicians had recommended them, and their parents accepted these decisions (IP16P; also refer to *Accountability*).

#### Use Experience

The participants reported that the period when they were diagnosed with diabetes was stressful, especially the early days after diagnosis, and that they had tried to learn about the disease and how to operate the devices (IP3P, IP4P, and IP5P). Subsequently, a self-management routine was established, and self-management became easier, especially for the participants who had used devices for longer or had been living with diabetes for an extended period (IP3P and IP6P):

She was only a baby...with rotavirus...that was the trigger...Took her to the doctors, he said “oh look...it could be diabetes”...I had no idea what he was talking about...He phoned me that night and he said “we need you down the hospital straight away.” And at that point, my life changed.

IP10P

The participants also reported trialing multiple devices until the best self-management solution was found to maintain blood glucose level in the ideal range (IP13 and IP15):

Before that he tried the Dexcom CGM, and before that he tried the...Medtronic Guardian one...it didn’t really work...so that’s why we went to the Dexcom, and then since he’s been on this new pump, then we went back to the Medtronic one.

IP15P

#### Facilitating Conditions

Health insurance and subsidy schemes were reported to impact device use and choice; there were waiting periods for chronic conditions and device replacements (IP2P, IP3P, IP7P, IP8P, IP13P, IP14P, IP15P and IP16P) and delays in technology release processes (IP5P, IP12P, and IP14P). The release of new pump features was welcome; however, at the same time, the parents expressed concerns about the effects of new features on self-management, such as those overriding basal adjustment (IP10P). There was a desire for improved funding options to pay for devices (IP5P). The high cost of devices—especially the insulin pump—was criticized by several participants (IP2P, IP4, IP5P, and IP7), which led to the fear of device breakage (IP5P) or attempts to extend a device’s life span (IP2P):

We had to wait until our health insurance covered it. Because they’re expensive. And so now we’re waiting to get the Medtronic sensor, because it’s covered with one of the rebates or whatever that the government do for people under 21, but it’s not covered for my older [child]...It’s very expensive, it’s thousands of dollars a year.

IP7P

Moreover, 12% (2/16) of participants mentioned problems with customer service, provided through a company hotline, for both CGM devices (IP2P) and insulin pumps (IP13P). In contrast to some difficulties with device customer service, most participants mentioned good hospital infrastructure with ongoing support provided by the diabetes health care team, including training on how to use an insulin pump (IP14):

That’s the staff at the Canberra Hospital, they’re brilliant...There’s the paediatric diabetes team at the Canberra Hospital...the diabetic educators. They’re great.

IP13P

A hospital hotline was available for the families, connecting them to diabetes educators or on-call registrars (IP4 and IP14P), as well as email contact with doctors, educators, and dietitians (IP14 and IP14P, IP15P and IP16P). Mobile numbers of endocrinologists were also provided (IP14P), and phone consultations in addition to face-to-face consultations were made possible during the COVID-19 pandemic (IP14P). The parents valued face-to-face support from the health care team (IP15P and IP16P). For some participants who had been treated in both rural and urban clinics, the rural clinics were reported to deliver less efficient consultations than urban clinics (IP2P and IP15P), and communication among the health care teams in a rural setting was criticized by one of the participants (IP15P). The interview data pointed to the possibility that the quality of diabetes support might also vary between general practitioners or pediatricians and diabetes specialists (IP13P and IP15P).

In addition to health care support, Facebook (Meta Platforms) groups for parents of children with T1DM (or other social media) were used for nonmedical queries (IP13P). Support from school personnel, such as teachers, was described as being important for safe diabetes management outside home. Most of the young participants with T1DM reported that their teachers were supportive of their device use and diabetes management in class (IP5, IP6P, IP7, IP8P, IP9P, IP13, IP13P, IP14, IP15, and IP16P); however, some highlighted problems with relief teachers (IP2 and IP4) or inappropriate behaviors by uninformed teachers or the school (IP3):

There was one time where a teacher asked to take the pump...well, one of my diabetic friends that goes to that school, he got his pump off him for the day, which probably wasn’t good.

IP4

School management plans regarding device use in school were agreed on together with the health care team (IP3P and IP14) in close cooperation with parents (IP6P). CGMs were perceived as particularly helpful in the school environment (IP16P).

### VSD Factors—System Features

#### Connectivity

Although the pump could not be directly connected to a phone for easier operation (IP14) and direct data sharing from the pump was not possible (IP2), most participants used a CGM to make the *“*devices...talk to each other*”* (IP14P; also reported by IP2, IP3P, IP4, and IP12). They tried to achieve a closed loop system, with partial success (IP7P), connecting the phone, CGM, and pump (IP2), whereas some used an Apple watch in addition (previous use: IP13 and IP13P; current use: IP6 and IP2). The Medtronic system (CGM plus pump) had the “suspend when low” function (IP2P and IP5P) but did not allow data sharing; by contrast, the Dexcom and pump combination allowed data to be shared with several other devices (IP7) but did not provide the suspend function (IP1 and IP5P). A combination of both was wished for by the participants (IP1P, IP5P, and IP9P). It was perceived as difficult to choose 1 pump or CGM system, as “they all have their pros and cons. A bit like Ford and Holden [cars]” (IP2P). Depending on the chosen system, patient data could be automatically accessed by the health care team for some patients, for example, through the Dexcom Clarity (IP4, IP13P, IP14, and IP16P), whereas others had to upload their data to a cloud system to share them with the health care team (eg, Medtronic CareLink, IP15P). Apart from the health care team, Dexcom data were mostly shared within the family (IP1P, IP4P, IP5P, IP7, IP12P, IP13, and IP14), particularly those of younger children (IP15P). The parents especially valued the sharing option (including alarms) as a “safety net” (IP1P, IP7P, IP9P, and IP16P). This option also made Dexcom the most popular CGM in the study sample (IP1).

#### Data Analysis, Retrieval, and Storage

The participants reported that their endocrinologists used the transmitted CGM data to calculate an average value resembling their hemoglobin A1c (HbA1c) level, especially when HbA1c testing was not possible (IP16P). However, this was described as “not always a hundred percent accurate” (IP14P). Moreover, the participants valued weekly summaries (IP4, IP7P, and IP14), data trends (IP1P and IP14), and other graphical output or visualization options of the CGM (IP5P).

To access or retrieve diabetes information, the parents used diabetes information websites (IP13P), Facebook groups for people with T1DM (IP1P and IP13P), and Google (IP4P). To access blood glucose data or information about food, young people used glucose tracking apps and food database apps (IP4):

So when I’m on my phone...I’ll quickly switch to that [app] and check it. So then when I turn on the phone, I just glance at it and do my business before I turn it off, I just check it again.

IP1

Data storage was reported as a feature of insulin pumps and blood glucose meters (IP6).

### VSD Factors—Values

#### Accessibility

Accessibility—the system’s availability, adaptability, and portability—was mentioned when the young people and their parents described situations in which technology required flexibility. This included diabetes management at night, during sports, at school, or when participating in sleepovers or camps. Devices facilitated attendance at camps or sleepovers (IP1P and IP15P), with CGM and its data sharing options being more useful than the pump (IP6P and IP9). Some young people kept their CGMs on their bodies during sports and swimming (IP7, IP12, and IP16), whereas some took it off only during swimming (IP6). Water resistance of the pump was mentioned by one of the participants (IP4). The device tapes came off at times (IP1, IP2P, IP5, IP7-8, and IP13-14), so better adhesives (IP1, IP3, and IP14P), as well as reduced device sizes to facilitate physical activity (CGM: IP1 and IP3; pump: IP6-7 “bulky”), were requested. The participants expressed a desire for devices that were small but still effective (IP4) and for fewer devices that a person is required to carry with them (IP1P and IP5P):

What is needed is an all in one device (CGM, insulin pump and control system) that doesn’t require tubes and can be controlled via an app with an algorithm that constantly regulates blood sugars that can operate as a closed loop system.

IP14P

Taking the pump off during swimming (IP11 and IP13) or sports (IP10 and IP15) initiated a “panic mode” that “you need to put it on silent otherwise your bag...is making all sorts of wonderful noises” (IP13P). Device alarms were reported to be challenging in various situations (IP9 and IP15P), such as when interfering with sleep (IP1P, IP2P, IP3P, IP12, and IP13P) or activities in school. Alarms were perceived as embarrassing in school (IP1, IP3, IP4, and IP15), which led to ignoring (IP2P, IP6P, and IP9) or limiting them (IP7 and IP15P) or turning the vibration or silent mode on (IP2-4 and IP15). Some parents tried to teach their children not to be ashamed of their devices. (IP2P). However, at night, alarms created a feeling of safety (IP1, IP3P, IP4P, and IP14), especially for the parents when the young people would sleep through them (IP1P, IP2, IP4, IP5P, IP6, IP7, IP8P, IP9, IP14P, and IP16P). However, alarms could be customized for different situations (IP1).

#### Accountability and Autonomy

At night, most parents reported taking care of their children’s diabetes management (IP2P, IP5P, IP7, IP8P, IP10P, IP13P, and IP16P), which is related to perceptions of accountability and autonomy [18]. One of the participants stated that responsibility lay with their parents at night (IP1). Commonly, the parents transferred part of the responsibility to their children when they became teenagers (IP2P and IP15P), assisting them when needed (IP2P and IP16P). At that stage, the adolescents preferred some independence from their parents and the freedom to make their own decisions (IP23P, IP3, IP5, IP9, and IP10P), as they felt more confident and in control of their diabetes devices (IP14):

I feel like you reach a point where we kind of know a bit more [than the doctor]...because we’re the ones experiencing it kind of every day.

IP14

Some adolescents felt like role models for younger children with T1DM (IP4P). In contrast to young people, some parents had problems letting go of the responsibility, wishing to continue data sharing (IP15P), which was at times perceived as intrusive by the young people (IP2), as they reported being fine without CGM data sharing (IP15). However, CGM data sharing also facilitated independence in some young people and reduced anxiety in parents when their control over the children was reduced (IP16P).

#### Trust

Independent management was associated with trust in the devices, which was affected by accuracy and device failures (IP8P). CGM technology was reported to be inaccurate at times (IP1, IP2, IP3P, IP8, IP14, IP14P, and IP16P), for example, when “it...wears down” (IP16P; similar: IP11) and when time lags occur (IP1, IP3P, IP10, IP11, IP12P, IP13P, and IP16P), whereas the pump was mostly accurate and reliable (IP3, IP8, and IP15). Technical device failures, such as blocked insulin tubing, were reported for both the pump (IP4, IP6, IP9, and IP15) and CGM (IP1-2, IP8P, IP10, and IP13). Most participants used finger pricking as a backup option when they were uncertain about the device accuracy or when recalibrating the device (IP1-6, IP8P, IP10, IP12, IP13P, IP14-15, and IP16P). They also considered other measures to improve the safety net, such as a diabetes assistance dog (IP1P). Device calibration was perceived as difficult at times; for example, taking paracetamol affected blood glucose readings and respective calibration (IP1-2 and IP14). Several participants mentioned that they trusted their bodies and the blood glucose meter more than the CGM devices (IP1, IP5, IP8, and IP14-15), for knowing when hyperevents or hypoevents are occurring (IP3, IP10, IP12, and IP13P). Trust in the health care team was equally relevant, as this gave the participants a feeling of safety in case they needed medical support. This was reported by almost all the participants (refer to *Facilitating Conditions*).

#### Sense Making

The participants stated that their ability to make sense of the data and give meaning to them increased with advancing age, disease duration, and independence. Diabetes education and device training played a crucial role in understanding data and managing diabetes independently (IP14 and IP16P). It was described as a gradual and individual process of learning how to best deal with the disease, its management, and device use (IP13). Graphical device outputs facilitated the sense making of numerical values (refer to *Data Analysis*). Management approaches were individual, and solutions had to be adapted to each patient, with no one-size-fits-all solution available (IP14 and IP15P). Some participants preferred multiple daily injections over an insulin pump (IP1 and IP16) or vice versa (IP6 and IP8), whereas others preferred the pump more than CGM (IP15), with CGM not working for some (IP3 and IP8).

#### Compliance

The degree of independence partly depended on the overall style of self-management between the parents and their children and the compliance with the care regimen. Some young people reported overmanagement (IP1 and IP1P, IP5), whereas others were not following care recommendations strictly (IP2). The omnipresence of the disease and the devices was reported as overwhelming by some participants who were strict in their management (IP5P); the participants reported that especially during puberty, it was difficult to control blood glucose levels (IP14, IP15P, and IP16P) and that they made use of the devices to improve self-care (IP14).

#### Dignity, Empathy, and Feedback

Negative self-management outcomes impacted the participants’ dignity related to their sense of pride and self-respect, for example, receiving unfair treatment because of diabetes. Some participants reported a sense of discrimination because of being unfairly treated at school (IP14P):

I was forced to go back in sickbay which I didn’t...want to go there because the stomach bug was there and that’s really bad for diabetics to get a stomach bug. So we had to actually go to the hospital and change my claim...that I am allowed to inject in class.

IP14

One of the parents said that “we had to go through a lot of steps [to use the CGM in class]...you sort of feel like there’s this constant discrimination for something that he has no control over...and [there are] safety concerns” (IP3P). In another situation *“*they sort of buddied them up for the first school camp...but I think they don’t have to be coupled just because they’ve got type 1 diabetes” (IP13P); especially young people's dignity could be impacted because of such treatments. Despite these challenges, empathy was reported—most young people explained that their friends, peers, and family members accepted their medical condition and were very supportive (IP1, IP5P, and IP13). Empathy was also expressed by the health care team when feedback and support were reported (refer to *Facilitating Conditions*). Parents or the health care team provided feedback based on data sharing, as well as devices in the form of automated feedback.

#### Hope and Joy

Overall, most participants hoped for and expected improvement in their self-management with the devices and tried to achieve normality in life, being able to enjoy life rather than being burdened by the omnipresence of the disease (IP5P, IP15P, and IP16P). Diabetes burnout was mentioned as a challenge with the omnipresence of diabetes technologies, including constant messages (IP4P) and the burden of wearing the pump all the time (IP5P). The participants reported high psychological pressure related to diabetes management, including anxiety (IP1, IP2P, IP4, IP8P, and IP14). The use of devices helped alleviate this anxiety, especially for parents (IP1P, IP2P, IP7, IP12P, and IP16P). Moreover, the participants tried to manage negative feelings such as discomfort, annoyance, and frustration related to device insertion and site changes (IP2, IP14, and IP16), carrying several devices (IP3, IP14P, and IP16), and operating the devices (IP3). In particular, pump tubing was mentioned as cumbersome (IP1, IP4, IP7, and IP9). Breath devices, such as breath ketone sensors, were considered a potentially interesting noninvasive alternative to reduce pain related to needles and finger pricking (IP1). New CGM and pump models were expected to solve these challenges (IP14), for example, with fewer calibration requirements (IP2P) or easier insertion expected in the new CGM models (IP16). Overall, the participants perceived that “benefits outweigh the negatives” (IP3P) regarding diabetes technologies.

#### Privacy

Surprisingly, privacy concerns or related aspects were not reported throughout the interviews, and none of the participants made any mention of data privacy.

Overall, the expectations of what devices should look like were mentioned throughout the interviews and were in accordance with all the theoretical factors from the models. Summarizing these expectations resulted in a list of device characteristics that meet young people’s needs (mainly related to CGM and insulin pump use), including specific features and designs, as presented in Table 4. These included, for example, improved reliability and accessibility of diabetes technologies, facilitated device interconnectivity, data sharing and fully automated closed loop systems, improved device algorithms, device noninvasiveness, and reduced device sizes and the number of devices to be carried.

**Table 4:** Device characteristics reflecting young people’s needs—derived from the interview findings and structured along theoretical factors.

Model, category, and factor		Device characteristics
**UTAUT^a^ core determinants**		
	Effort expectancy	Improved ease of use of devices Reducing effort to use technology Facilitated integration in everyday life
	Facilitating conditions	Improved device infrastructure: easy to access customer service, reduced device cost or improved funding or subsidies, and quicker release of new technology and improved access to this advanced technology (shorter waiting periods) Improved training related to device use in school and family environments Facilitated cooperation with the health care team
	Performance expectancy	Features to make technology-supported self-management easier Facilitated decision-making to select devices (eg, pump brands) Taking preferences and expectations into account through personalization features Increased communication of success in self-management (eg, positive feedback and rewards)
	Social influence	Improved education on device selection
**UTAUT moderators**		
	Gender and age	Devices taking the age of patients into consideration (the needs of young children are different from those of adolescents, eg, regarding autonomy in self-management)
	Use experience	Technology features adaptable to the needs of patients who were newly diagnosed versus patients with long disease management experience Personalization
	Voluntariness of use	Features related to the accountability or autonomy of young person with diabetes Avoiding extreme controlling mechanisms and offering some flexibility for the individual in data sharing setups, etc
**VSD^b^ system features**		
	Connectivity	Improved connectivity among CGM^c^, pump, and phones (closed loop), especially connecting pump directly to phone (without the need of CGM) Fully automatized system Improved data sharing possibilities, including no need to download data before sharing, and quick data access for HCPs^d^ and caregivers (with opportunities for independence in adolescents; refer to the Accountability/autonomy category) Combination of data sharing and automatized device cutoff mechanisms when blood glucose level is low Improved connectivity with other devices (eg, smart watches) Personalized regulation of device feedback (alarms and notifications)
	Data analysis	Improved algorithms and result display of insulin pumps Improved visualization of results Data prediction
	Data retrieval and storage	Facilitated data retrieval (eg, nutritional information included in device platform) and data storage (automatic storage of data, eg, regarding physical exercise) Facilitated interconnection to other apps and websites
**VSD values**		
	Accessibility	Devices automatically adapting to new situations and conditions (eg, travel, sports, camp, sleepover, and night) Facilitated data accessibility in these situations, including reduced device size, improved charging possibilities, robust devices, waterproof devices, improved device adhesives, and improved alarm settings (personalization and reducing faulty and excessive alarms) Facilitated data sharing Improved cutting off when blood sugar level is low
	Accountability and autonomy	Features supporting increased self-responsibility and independence in adolescents, with options for facilitated data sharing with HCPs and caregivers (potentially giving youth the opportunity to decide when data are not to be shared) Facilitated diabetes management at night (number of alarms, etc) Improved parent-child dynamics
	Compliance	Features that improve compliance with care regimen and reduce overmanagement at the same time
	Dignity	Features that reduce discrimination or unfair treatment, devices improved for use in public or at school (alarms, injection in class, etc)
	Empathy	Features to share empathy Improved communication features
	Feedback	Facilitated feedback from HCPs and caregivers through the devices Improved automated and personalized feedback (without increasing the number of messages and input, which might lead to diabetes burnout, for example, by providing personalization options for notifications)
	Hope and joy	Features that enable normality in life, reduce the omnipresence of disease and device overload, and reduce anxiety (feeling of safety) Reduced alarms and messages to prevent diabetes burnout (personalization) Reduced discomfort with devices, for example, reduced number of devices to be carried, reduced insertion discomfort, noninvasiveness, improved tapes, and no use of tubes and wires (pump)
	Privacy	Data privacy of sensitive health data
	Sense making	Data that can be easily understood and interpreted, including by youths Graphical outputs for fast interpretation
	Trust	Accuracy and reliability of devices without time lags, mirroring hypoglycemia and hyperglycemia awareness, reduced technological failures, and facilitated calibration or lack of need for calibration (increasing trust)

^a^UTAUT: unified theory of acceptance and use of technology.

^b^VSD: value-sensitive design.

^c^CGM: continuous glucose monitor.

^d^HCP: health care professional.

## Discussion

### Principal Findings

All the factors in the UTAUT and VSD theories, except for one (privacy), aligned with the themes independently identified through data-driven user experience analysis, indicating that these theories have value in structuring the data analysis and empirical findings. This also demonstrates the alignment of the empirical interview data with both the existing theoretical models. Multimedia Appendix 3 summarizes the alignment of the initial themes with the theoretical factors and provides exemplary data excerpts.

We were intrigued that the participants in our study did not raise issues of privacy, as this was considered to be of great importance in previous research examining the VSD of technologies [27]. Britton and Britton-Colonnese [38], for example, highlighted the data privacy and security risks associated with CGMs, such as the lack of possibilities to control how patient data are collected, stored, and used. Young people are more likely to be concerned about privacy on the internet than older people, recognizing the compromises that they must make to their own privacy to use embedded web-based networks [39]. It is possible that CGM data do not strike young people as compromising privacy as clearly as social media does. Future user experience research should focus specifically on privacy aspects to elucidate potential concerns of young people and their parents regarding diabetes technologies—such as if CGM data are regarded as risky for privacy—and how these concerns might be important for device design.

We compared this study’s findings with a previous systematic integrative review of 17 studies on the experiences of young people living with T1DM and their caregivers with using technologies to manage T1DM [8]. The review identified eight themes: (1) expectations of the technologies before use, (2) perceived impact of technology use on sleep and overnight experiences, (3) experiences with alarms, (4) impact of technology use on independence and relationships, (5) perceived impact of technology use on blood glucose control, (6) device design and features, (7) financial cost, and (8) user satisfaction. Despite the independent analysis of both studies, there was a major overlap between the review themes and our UTAUT- and VSD-aligned interview study findings (Tables 1 and 2). Our results confirmed the results of previous studies, which we see as an important research strategy to validate empirical results.

Messer [40] argued that with new technological advancements, expectations among some individuals regarding new diabetes technologies are high at first (idealism) but then fall when reality does not match these expectations. The systematic review [8] reported that some of these expectations are related to the self-sufficiency of these technologies, resembling an actual artificial pancreas system that can make life easier and enable normality, reducing the burden of the disease. Similar wishes and expectations were expressed in the interviews, for example, related to fully automatized systems (factor *Connectivity*). In line with the systematic review [8], the participants in our study indicated that reality diverted from these expectations, with inaccuracy problems reported in CGMs (time lag in interstitial fluid measurements) and technical failures occurring in both CGMs and insulin pumps. When reality does not match initial expectations, it can lead to a risk of nonadherence and discontinuation of therapy due to frustration [40]. By contrast, not all users initially set their expectations high, as shown in another study by Quintal et al [41], with some people expecting inconveniences regarding technical limitations, cost, wearability, or similar aspects before using the technologies [41]. Overall, accuracy and reliability were highlighted as the most important technological criteria in our study, in line with other studies [8,42].

Apart from expectations before use, diabetes management at night and device alarms, as found among the review themes [8], were major concerns for the participants in our study (factor *Accessibility*), whereas independence was a topic especially raised by adolescents or teenagers (factor *Accountability/autonomy*) in both our study and the review. Similarly, Babler and Strickland [43] found that adolescents experienced challenges with independent care and conflicts with their parents. Diabetes-related distress, family conflict, and depressive symptoms were reported as barriers toward using diabetes technologies [44]. Previous research described a learning curve traversed by individuals newly diagnosed with T1DM as they gradually learn how to self-manage T1DM with devices and in cooperation with important others such as the health care team and parents [45]. Distress was mentioned in our study as being particularly high in the early days after the diagnosis. Both the review [8] and our study reported that diabetes technologies were able to alleviate psychological challenges such as anxiety to some extent.

The outcomes of technology use for self-management and overall satisfaction with the devices were discussed as part of the UTAUT factors *Performance and Effort Expectancies* and VSD values in our study, with most participants acknowledging the benefits of the devices. A previous study on CGM and insulin pump use in the United States and Germany [46] stated that 47% of pump users were very satisfied with the pump and 98% would recommend the pump to others, whereas only 84% would recommend CGM to others. Apart from device failures and in line with the review [8], the participants in our study reported that cost and funding were major barriers to device accessibility.

Finally, our study participants highlighted certain aspects that expanded the themes of the systematic review [8]. These included perceived discrimination towards having a chronic disease such as T1DM. This was a good fit for the VSD factor *Dignity*. In contrast to a previous study showing difficulties in integrating technologies into clinical workflows [42], most participants in our study reported the process of sharing their diabetes data with the health care team and integration of these data into a consultation to be smooth. According to Vrijhoef et al [47], integrated care pathways could be used for mutual decision-making between patients and health care professionals, supported by information technologies that facilitate patient empowerment and improve monitoring and management [47]. Overall, one particular strength of our study was the combination of data-driven and theory-driven analyses. None of the 17 studies included in the systematic review [8] used a theoretical foundation to underpin their examination of experiences, despite the proven benefit of using theory in research [15]. Incorporating knowledge from 2 different theoretical approaches (technology acceptance and technology design) into our study design enabled us to produce research aligned with a theoretical foundation and add new (knowledge from) empirical data to the existing theories. This has resulted in a piece of research that supports the use of theory in user experience research, suggesting that such an approach is fruitful for the future; this is because theory can inform our user experience data analysis, and new empirical data can be provided to support or expand the existing theoretical foundations. In our study, all the interview themes could be aligned with the theoretical factors from UTAUT and VSD, suggesting that the 2 theories provide a comprehensive foundation (using UTAUT alone would have made it difficult to align emotional themes such as discrimination, as they do not align with UTAUT factors). The combination of UTAUT with VSD allowed us to combine 2 theoretical approaches examining technology and its uptake from different angles. This has the potential to expand the focus of research on one topic, by taking 2 lenses into consideration. Similar approaches for combining theories can be found in recent literature on various health topics [48,49]. The minimal overlap of factors in our 2 selected approaches, the difference in focus on technology and its uptake, and the possibility to fill the theoretical limitations of one theory with the other, as described above, means that the 2 approaches complement each other very well. Thus, a sound foundation is available for understanding user experiences to advance diabetes technologies. Further research is needed on such a hybrid approach to further evaluate and substantiate the use of theory combinations in empirical research. This will ultimately inform the design of new technologies and addresses a general lack of theoretical underpinnings in studies on diabetes and other health technologies [14].

### Study Limitations

Our findings were based on the self-reports of young people with T1DM and their caregivers. Additional perspectives of health care professionals would also provide valuable insights into this topic. A degree of self-selection of the participants was unavoidable because of the voluntary nature of study participation. This might have led to an overrepresentation of young people with T1DM who managed their disease well. Perspectives might differ in people with T1DM who struggle with its management or who do not follow their care regimen. However, we did not have access to the participants’ clinical results, such as HbA1c, to confirm how well their diabetes was managed. We did not aim to quantify the results; thus, the results are not generalizable and specifically correspond to young people in the respective setting. However, we found a large overlap of our study findings with other studies’ results, as shown in the comparison with a recent systematic review [8]. To quantify the results or eliminate differing technical properties of the various insulin pumps or CGMs, a study with a larger sample would be required.

### Conclusions

Our study indicates that technologies for diabetes self-management require continual advancement to meet the needs and expectations of young people with T1DM. Understanding their experiences and challenges with using devices enabled us to identify a variety of device characteristics that reflect the needs of the young people interviewed. The identified characteristics can be useful in designing and developing improved technologies, ideally including participatory design approaches. Our research highlights the benefits of the transdisciplinary use of exploratory and theory-informed methods for designing improved technologies. In our study, theoretical technology acceptance and VSD approaches proved useful as a combined foundation for structuring the study findings regarding technological experiences. Our results confirmed the results of previous studies and that the combination of theory and empirical results can offer greater surety. In addition to clinical or regulatory guidelines, the use of theories is important to integrate new empirical findings into the existing theoretical knowledge and expand and further develop theoretical knowledge to advance the rigorous and informed design of diabetes technologies.

## Appendix

Multimedia Appendix 1 Initial themes derived from the interviews (coding schema).

Multimedia Appendix 2 Additional value-sensitive design factor explanations.

Multimedia Appendix 3 Alignment of the initial themes with theoretical factors and relevant data excerpts.

Multimedia Appendix 4 COREQ (Consolidated Criteria for Reporting Qualitative Research) checklist.

## Abbreviations

CGM: continuous glucose monitor
COREQ: Consolidated Criteria for Reporting Qualitative Research
FGM: flash glucose monitor
HbA1c: hemoglobin A1c
IP: interview participant
T1DM: type 1 diabetes mellitus
UTAUT: unified theory of acceptance and use of technology
VSD: value-sensitive design

## Data Availability

Further information about the study can be provided upon request (corresponding author).
