# Supplementary material for: Toward Diabetes Device Development That Is Mindful to the Needs of Young People Living With Type 1 Diabetes: A Data- and Theory-Driven Qualitative Study
Source: JMIR Diabetes. 2023 Jan 25;8:e43377. doi: 10.2196/43377 (PMC9947809; doi:10.2196/43377)
Supplement: Multimedia Appendix 2 [file diabetes_v8i1e43377_app2.docx]

Appendix 2. Additional Value-Sensitive Design Factor Explanations

| **Model: category** | **Factor** | **Factor explanation/definition**  **Friedman et al** [27] | **Factor explanation /definition applied to self-management of diabetes in adults as identified by Dadgar and Joshi** [18] | **Factor explanation / definition applied to self-management of type 1 diabetes in young people adapted from Dadgar and Joshi** [18] |
| --- | --- | --- | --- | --- |
| VSD: system features | Connectivity |  | The system features that enable a diabetic patient to interact and share information with healthcare providers, family members, friends, and other diabetic patients. This category allows patients to develop and/or maintain a network of people who can interact, communicate, and support the practice of SM in and through a digital device (derived from study). | Features that allow the user to interact with the devices and share information with others |
|  | Data analysis |  | The system features that enable a diabetic patient to make sense of diabetes data over time. These features allow patients to manipulate and visualize data (e.g., effective representations of past trends and patterns) and augment the capacity to process and apply insights from data in establishing the dynamic and continual process of self-regulation and adjustment crucial to managing a chronic condition (derived from study) | Features that allow the user to make sense of data over time |
|  | Data retrieval and storage |  | Data retrieval:  The system features that enable a diabetic patient to access data. These features allow patients to obtain pieces of information to continually monitor their condition in real time to trigger a response (cognitive, emotional, or behavioral) necessary for maintenance of a chronic condition (derived from study).  Data storage:  The system features that enable a diabetic patient to store, log, and track the diabetes data (derived from study). | Features that allow the user to access and store data |
| VSD: values | Accessibility |  | The properties of being available when needed (derived from study) | The system’s availability, adaptability and portability |
|  | Accountability and autonomy | Accountability:  Refers to the properties that ensures that the actions of a person, people, or institution may be traced uniquely to the person, people, or institution  Autonomy:  Refers to people’s ability to decide, plan, and act in ways that they believe will help them to achieve their goals | Accountability:  The properties that ensure that the actions of an entity may be traced uniquely to that entity (adapted from [30])  Autonomy:  Patients’ ability to decide, plan, and act independently in ways that they believe will help them achieve their goals (adapted from [30]) | Self-responsibility for habits and care performance, with independent behavior and decision making |
|  | Compliance |  | Adherence to activities such as taking drugs, using medical devices and ICTs for selfcare and self-directed exercises (derived from study) | Adherence, following diabetes care plan |
|  | Dignity | Aligns with “Freedom from bias”  Refers to systematic unfairness perpetrated on individuals or groups, including pre-existing social bias, technical bias, and emergent social bias | A sense of pride in oneself and self-respect. It captures both the positive and negative consequences of preserving dignity. On one hand, good SM provides a sense of pride in controlling chronic problems, and on the other hand, it impedes SM as patients try to withhold information or not seek help in an attempt to preserve their pride (adapted from [31] and [32]). | Sense of pride and self-respect (impacted by negative outcomes or unfair treatment for performance) |
|  | Empathy | Aligns with “Courtesy”  Refers to treating people with politeness and consideration | The ability to understand and share the feelings of diabetic patients (derived from study). | Desire to be understood by others |
|  | Feedback |  | Information about patient’s SM activities used as a basis for reminders, improvement, or positive reinforcement (derived from study). | Responses by others or technology |
|  | Hope and joy | Aligns with “Human welfare”  Refers to people’s physical, material, and psychological well-being | Hope:  A patient’s motivation to achieve future-oriented expectations and personally valued goals which will give meaning and depend on personal activity or characteristics (adapted from [33]).  Joy:  The feeling of pleasure (derived from study). | Motivation to achieve future-oriented expectations and personally valued goals, also including joy in life |
|  | Privacy | Refers to a claim, an entitlement, or a right of an individual to determine what information about himself or herself can be communicated to others | A claim, an entitlement, or a right of an individual to determine what information about himself or herself (e.g., medical data, taking medication in public, being a diabetic) can be communicated to others (adapted from [30]). | Information protection when sharing sensitive (health) data |
|  | Sense-making |  | The ability to give meaning to data that captures patients’ SM activities and behaviors (derived from their study). | Ability to give meaning to data |
|  | Trust | Refers to expectations that exist between people who can experience good will, extend good will toward others, feel vulnerable, and experience betrayal | Expectations (of reliability, truth, and/or the ability to do the right thing) that exist between people and/or technology (adapted from [30]). | Trust in technology, oneself, and others |
